# Systematic Review and Meta-analyses of the Effect of Chemotherapy on Pulmonary Mycobacterium abscessus Outcomes and Disease Recurrence

**DOI:** 10.1128/AAC.01206-17

**Published:** 2017-10-24

**Authors:** Jotam G. Pasipanodya, Deborah Ogbonna, Beatriz E. Ferro, Gesham Magombedze, Shashikant Srivastava, Devyani Deshpande, Tawanda Gumbo

**Affiliations:** a Center for Infectious Diseases Research and Experimental Therapeutics (CIDRET), Baylor Research Institute, Dallas, Texas, USA; b Department of Public Health and Community Medicine, Universidad ICESI, Cali, Colombia; c Department of Medicine, University of Cape Town, Observatory, Cape Town, South Africa

**Keywords:** macrolides, Mycobacterium abscessus, hollow-fiber model, medical outcomes, pulmonary infection

## Abstract

In pharmacokinetic/pharmacodynamic models of pulmonary Mycobacterium abscessus complex, the recommended macrolide-containing combination therapy has poor kill rates. However, clinical outcomes are unknown. We searched the literature for studies published between 1990 and 2017 that reported microbial outcomes in patients treated for pulmonary M. abscessus disease. A good outcome was defined as sustained sputum culture conversion (SSCC) without relapse. Random effects models were used to pool studies and estimate proportions of patients with good outcomes. Odds ratios (OR) and 95% confidence intervals (CI) were computed. Sensitivity analyses and metaregression were used to assess the robustness of findings. In 19 studies of 1,533 patients, combination therapy was administered to 508 patients with M. abscessus subsp. abscessus, 204 with M. abscessus subsp. massiliense, and 301 with M. abscessus with no subspecies specified. Macrolide-containing regimens achieved SSCC in only 77/233 (34%) new M. abscessus subsp. abscessus patients versus 117/141 (54%) M. abscessus subsp. massiliense patients (OR, 0.108 [95% CI, 0.066 to 0.181]). In refractory disease, SSCC was achieved in 20% (95% CI, 7 to 36%) of patients, which was not significantly different across subspecies. The estimated recurrent rates per month were 1.835% (range, 1.667 to 3.196%) for M. abscessus subsp. abscessus versus 0.683% (range, 0.229 to 1.136%) for M. abscessus subsp. massiliense (OR, 6.189 [95% CI, 2.896 to 13.650]). The proportion of patients with good outcomes was 52/223 (23%) with M. abscessus subsp. abscessus versus 118/141 (84%) with M. abscessus subsp. massiliense disease (OR, 0.059 [95% CI, 0.034 to 0.101]). M. abscessus subsp. abscessus pulmonary disease outcomes with the currently recommended regimens are atrocious, with outcomes similar to those for extensively drug-resistant tuberculosis. Therapeutically, the concept of nontuberculous mycobacteria is misguided. There is an urgent need to craft entirely new treatment regimens.

## INTRODUCTION

Mycobacterium abscessus complex members are rapidly growing mycobacteria associated with a wide spectrum of disease in humans, of which pulmonary disease is the most recalcitrant (1). M. abscessus complex members are ubiquitous in the environment, including in household tap water and bioaerosols (2, 3). Hospital water supplies have been linked to M. abscessus complex disease outbreaks (4, 5). Whole-genome sequencing (WGS) has also suggested a potential for person-to-person transmission (4). Two groups of vulnerable patients are most affected by M. abscessus complex: nonsmoking women of European descent who are >60 years old and have no history of lung disease and younger men <40 years old with prior lung disease, such as α-1 antitrypsin deficiency and cystic fibrosis (6). In the latter group of patients, M. abscessus complex can be a coinfection with other mycobacteria, leading to a high rate of disease recurrence (7). This makes diagnosis and microbial killing of the individual mycobacterial species difficult, given the differences in susceptibility between species (8, 9). Fortunately, advances in medical therapy have increased, but this has had the effect of increasing the proportion of the population at risk; thus, the disease numbers from M. abscessus complex now surpass those for tuberculosis in some places (6). Greater efforts toward improving quality of life for those affected are now warranted (10, 11).

M. abscessus complex has three subspecies: M. abscessus subsp. abscessus, M. abscessus subsp. massiliense, and M. abscessus subsp. bolletii (12). M. abscessus complex subspecies are naturally resistant to many antibiotics and rapidly develop acquired drug resistance (ADR), leading to the moniker “the antibiotic nightmare” (1). There are also differences in susceptibility to macrolides (clarithromycin or azithromycin), aminoglycosides, quinolones, and tigecycline between the subspecies, with better susceptibility seen with M. abscessus subsp. massiliense than M. abscessus subsp. abscessus (8, 9, 13). Thus, treatment of M. abscessus subsp. abscessus disease has a disadvantage of poor MICs from the beginning. Treatment guidelines recommend a regimental backbone of a macrolide, a β-lactam (cefoxitin, imipenem, or meropenem), and an aminoglycoside given in the first 2 to 4 months (6). The optimal duration of therapy is undefined. These drugs and their doses were chosen based on their use in other bacterial infections. Except for clarithromycin, drug sensitivity testing (DST) for the antibiotics used against M. abscessus complex is known not to predict clinical outcomes (14, 15). Examination of the recommended antibiotics as monotherapy, or as combination therapy, in the novel hollow-fiber model of pulmonary M. abscessus subsp. abscessus disease identified a biphasic response that was universally terminated by emergence of ADR, even at optimized doses not tolerable in patients (9, 13, 16–18). This led us to ask what the real response rates of the recommended regimen in the clinic are. Is the regimen any good? On the other hand, if a drug such as amikacin or the three-drug combination is associated with therapy failure and ADR even at maximum dose or exposure, then it cannot be improved upon (drugs or combinations cannot kill any more than their maximum possible kill) even if we changed the administration routes. Therefore, we systematically reviewed the literature, rated risk of bias, and then determined the proportion of patients attaining sustained sputum culture conversion (SSCC) and disease recurrence in clinical studies that examined different therapy regimens for pulmonary M. abscessus complex.

## RESULTS

### Study selection and patient characteristics.

**FIG 1:**
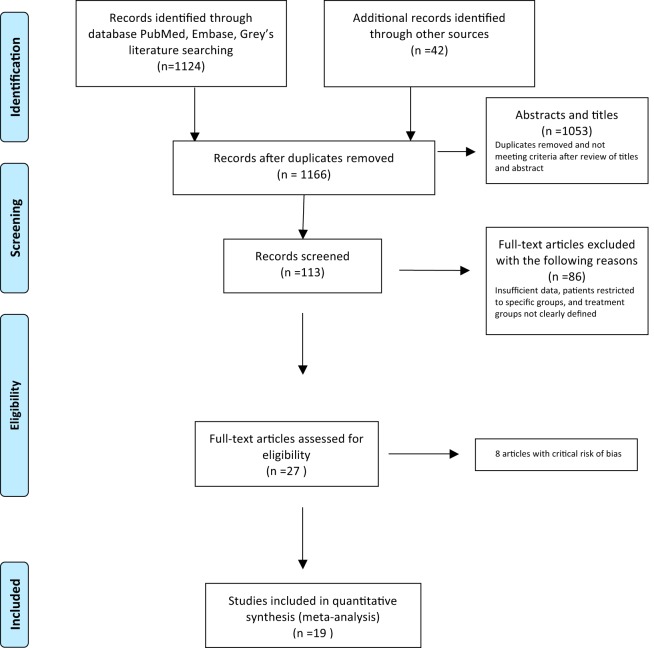
Study enrollment.

Of the 1,166 unique citations identified through systematic search, 27 were eligible for full review, and 19 of these were included in our analyses (Fig. 1) (4, 6, 7, 11, 12, 14–16, 19–36). We excluded 8 studies that we found to be at critical risk of bias: 4 studies did not clearly state therapy regimens, and the remaining 4 studies enrolled selected patient groups or the enrolled number of patients was <10, which was considered inadequate (see the supplemental material). Nonetheless, of the 19 selected studies, only 7 (37%) were at low risk of bias based on ROBINS-I (for risk of bias in nonrandomized studies–of interventions) criteria. The remainder, 12/19 (63%), were at moderate or serious risk of bias due to missing data across all domains and due to confounding (see Fig. S1 in the supplemental material). Eleven (58%) of the 19 studies were from Northeast Asia (2 from Japan, 8 from South Korea, and 1 from Taiwan), 7 (37%) were from North America, and one (5%) was from the Netherlands.

The 19 selected papers reported 1,533 immunocompetent pulmonary M. abscessus complex patients, of whom 1,013 (66%) were started on antimicrobial combination chemotherapy (Table 1). Of the 1,013 treated patients, M. abscessus subsp. abscessus was isolated in 508 (50%) patients, M. abscessus subsp. massiliense in 204 (20%) patients, and M. abscessus subspecies bolletii in only 3 patients (<1%). The remainder, 301 (30%), did not have isolates identified by subspecies and were termed M. abscessus with no subspecies specified. Three patients had mixed infections, i.e., had more than one subspecies (Table 2).

**TABLE 1:** Characteristics of pulmonary Mycobacterium abscessus complex studies identified through systematic review

Reference	Enrollment period	Study location (referral)	Study design^*a*^	No. of patients	Age, yr (range)	Male/female ratio	Outcome(s) examined^*b*^	No. (%) who died on therapy^*c*^^,^^*f*^
Macrolide-free regimens								
Griffith et al. (21)	1976–1991	Texas (southern USA)	RetroCS	120	54 ± 19.6	42:78	SCC, relapse, death	20 (17)
Macrolide-containing regimens								
van Ingen et al. (32)	1999–2005	Netherlands	RetroCS	30	53 (1–89)	19:11	SCC, death	4 (8)
Jeon et al. (14)	2000–2007	Seoul, South Korea	RetroCS	188	55 (43–67)	48:140	SCC, relapse, death (RSS)	2 (1)^*d*^
Jarand et al. (23)	2001–2004	Colorado, USA (36 states)	RetroCS	107	60.2 (20–85)	18:89	SCC, relapse, death	17 (16)
Lyu et al. (26)	2003–2008	Seoul, South Korea	RetroCS	112	53.2 (22–77)	10:31	SCC, relapse, death	0
Koh et al. (15)	2004–2008	Seoul, South Korea	RetroCS	145	57.6 ± 13.0	37:108	SCC, relapse (RSS)	0
Harada et al. (22)	1990–2010	Japan (12 centers)	RetroCS	102	68 (27–94)	39:58^*e*^	SCC, relapse (radiographic)	0
Tung et al. (31)	2006–2012	Kaohsiung, Taiwan	RetroCS	106	64.56 ± 14.11	44:62	SCC, relapse, death (radiographic)	15 (14)
Griffith et al. (7)	2000–2012	Texas, USA	RetroCS	21	75.5 ± 8.5	2:19	SCC, relapse	0
Namkoong et al. (27)	2004–2013	Tokyo, Japan	RetroCS	92	63.6 ± 8.5	2:11	SCC (radiographic)	NA
Koh et al. (25)	2007–2012	Seoul, South Korea	ProspCS	71	57 (50–64)	10:61	SCC (RSS)	NA
Czaja et al. (20)	2009–2012	Colorado, USA (southern states)	ProspCS	53	65 ± 11	7:40	SCC (RSS)	NA
Koh et al. (24)	2002–2012	Seoul, South Korea	ProspCS	67	57 (48–64)	15:52	SCC (RSS)	NA
Park et al. (30)	2006–2015	Seoul, South Korea	RetroCS	113	64 (52–71)	39:71	SCC (RSS)	NA
Macrolide-containing plus Investigational drugs in refractory disease^*g*^								
Olivier et al. (29)	2003–2010	Maryland, USA	RetroCS of inhaled amikacin	23	56 ± 16	4:16	SCC	NA
Wallace et al. (33)	2002–2006	Texas, USA	ProspCS of tigecycline	36	35.2 ± 22.2	7:29	SCC	NA
Yang et al. (34)	2013–2015	Seoul, South Korea	RetroCS of clofazimine	42	60 (53–69)	9:33	SCC (RSS)	NA
Olivier et al. (28)	2012–2015	North America (Canada and USA)	RCT of liposomal amikacin (NCT01315236)	90	58.5 ± 15.83	11:78	Semiquantitative mycobacterial growth scale, SCC, 6-min walk, adverse events	2 (2.2)
Choi et al. (19)	2005–2015	Seoul, South Korea	RetroCS	15	57 (48–67)	5:10	SCC, death	5 (33)
								

a RCT, randomized control trial; ProspCS, prospective cohort study; RetroCS, retrospective cohort study.

b SCC, sputum culture conversion; RSS, radiographic, symptomatic response.

c NA, data not available.

d Studies that only reported deaths due to pulmonary Mycobacterium abscessus complex disease in treated patients.

e Some data of patients with Mycobacterium abscessus subspecies bolletii are missing.

f Data analyzed or available in text or tables only.

g Refractory means failing initial therapy.

**TABLE 2:** Combination antimycobacterial chemotherapy and other clinical interventions examined by selected studies^*a*^

Reference	No. of patients treated	Type of infection^*c*^	*erm*(41) gene deletion (no. of patients/total no.)	No. of patients with CF, AAT, or CD^*b*^	Macrolide(s) used	Aminoglycoside(s) used^*d*^	Other antibiotics used in combination therapy; no. of patients who received surgery	Duration, mo^*e*^ (range)	
								Therapy	Follow-up
Macrolide-free regimens									
Griffith et al. (21)	120	120 NSS	NA	9 CF	None	i.v. AMK, daily	FOX, IPM, SXT, ERY, other anti-TB drugs; 7	NA	58.8 ± 4.8
Macrolide-containing regimens									
van Ingen et al. (32)	12	9 M. abscessus subsp. abscessus, 2 M. abscessus subsp. massiliense, 1 M. abscessus subsp. bolletii	NA	4 CF	CLR	i.v. AMK, daily	FOX, IPM, SXT, LVX, and first-line anti-TB drugs; 1	13	NA
Jeon et al. (14)	86	86 NSS	NA	NA	CLR	1/12 i.v. AMK, twice daily	FOX, IPM, CIP, DOX; 14	24.4 ± 0.2	12 (5–30)
Jarand et al. (23)	107	107 M. abscessus subsp. abscessus	NA	25 CF, 1 AAT	AZM, CLR	3/12 i.v. AMK	FOX, IPM, LVX, SXT and others, individualized based on DST; 24	52 ± 40.6	34 ± 21.1
Lyu et al. (26)	41	41 NSS	NA	NA	AZM, CLR	8/12 i.v. AMK, once daily	FOX, IPM, DOX, quinolones (CIP, MXF); 13	17.03 (5.46–41.63)	14.83 (0–48.1)
Koh et al. (15)	67	30 M. abscessus subsp. *Abscessus*, 37 M. abscessus subsp. massiliense	0/19 M. abscessus subsp. abscessus, 28/28 M. abscessus subsp. massiliense	NA	CLR	1/12 i.v., AMK twice daily	FOX, IPM, DOX, CIP; 0	23.1 ± 12.9 M. abscessus subsp. abscessus, 21.6 ± 7.7 M. abscessus subsp. massiliense	NA
Harada et al. (22)	64	42 M. abscessus subsp. abscessus, 20 M. abscessus subsp. massiliense, 2 M. abscessus subsp. bolletii	NA	NA	AZM, CLR, ERY	i.v. STP, AMK, KAN	FOX, IPM, DOX, quinolones (CIP, MXF); anti-TB drugs; 6	33 (3–178) M. abscessus subsp. abscessus, 36 (1–122) M. abscessus subsp. massiliense, 36 (4–68) M. abscessus subsp. bolletii	25 (1–120) M. abscessus subsp. abscessus, 18 (1–62) M. abscessus subsp. massiliense
Tung et al. (31)	56	56 M. abscessus subsp. abscessus	NA	NA	CLR	i.v. AMK	FOX, IPM, MEM, DOX, quinolones (CIP, MXF); anti-TB drugs; 0	12	NA
Griffith et al. (7)	11	11 M. abscessus subsp. abscessus	NA	NA	AZM, CLR	i.v. AMK	FOX, IPM	NA	48.3 ± 28.7
Namkoong et al. (27)	13	13 M. abscessus subsp. abscessus	NA	NA	CLR	i.v. AMK, thrice weekly	Faropenem, sitafloxacin, MIN, IPM; 0	21.31 ± 2.10	12
Koh et al. (25)	71	71 M. abscessus subsp. massiliense	16/16	NA	AZM, CLR	i.v. AMK	2-wk regimen of FOX, IPM; 3; 4-wk regimen of FOX, IPM, quinolones (CIP, MXF); 2	2-wk regimen, 15.2 (12.7–18.1); 4-wk regimen, 23.9 (23.1–24.1)	2-wk regimen, 14.7 (0.5–29.5); 4-wk regimen, 33.8 (12.3–50.3)
Czaja et al. (20)	47	47 M. abscessus subsp. abscessus	NA	9 CF, 5 AAT	AZM	9/12 i.v. and inhalation AMK daily	FOX, IPM, quinolones, CFO; 16	17.3 ± 6.6	24.97 ± 1.40
Koh et al. (24)	67	67 M. abscessus subsp. abscessus	7/44	NA	AZM, CLR	i.v. AMK	FOX, IPM, quinolones (CIP, MXF), DOX; 9	>12 mo	11.8 (3.6–27)
Park et al. (30)	113	56 M. abscessus subsp. abscessus, 54 M. abscessus subsp. massiliense, 3 mixed	27/56 (M. abscessus subsp. abscessus), 3/54 (M. abscessus subsp. massiliense)	NA	AZM, CLR	i.v. AMK, 3–5 times weekly	FOX, IPM; 5 (3 M. abscessus subsp. abscessus, 2 M. abscessus subsp. massiliense)	15.25 (7–29) M. abscessus subsp. *Abscessus*, 21.75 (16–30) M. abscessus subsp. massiliense	42.13 ± 22.47
Macrolide-containing regimen plus investigational drugs									
Olivier et al. (29)	15	10 M. abscessus subsp. abscessus, 5 M. abscessus subsp. massiliense	11/15	2 CF, 1 CD	CLR	Nebulized AMK	AMK nebulized 250 mg/ml daily; CLR given daily	60 (6–190)	19 (1–50)
Wallace et al. (33)	34	34 NSS		22 CF	AZM, CLR	AMK, tobramycin	FOX, IPM, MEM, quinolones (CIP, MXF), EMB, SXT; 3	8.5 ± 8.86	NA
Yang et al. (34)	42	42 M. abscessus subsp. abscessus	7/42	NA	AZM	4/52 i.v. AMK daily	Initial CLO therapy of FOX, IPM; 1; salvage CLO therapy added to existing regimen (quinolones [CIP, MXF], FOX, IPM); 2	Both treatment groups, 48.0 (24.8–48.0)	12.0
Olivier et al. (28)	32	32 NSS	NA	14 CF	AZM, CLR	3/12 liposomal AMK daily, Tobramycin	15 patients on intervention regimen of FOX, IPM, MEM, quinolones (CIP, MXF, LVF), DOX, linezolid, CLO, anti-TB drugs; 0; 17 patients on placebo regimen of FOX, IPM, MEM, quinolone (CIP, MXF, LVF), DOX, linezolid, CLO, TGC, anti-TB drugs; 0	>24	12
Choi et al. (19)	15	15 M. abscessus subsp. massiliense (all macrolide resistant)	14/15	NA	AZM, CLR	i.v. and inhalation AMK daily	FOX, IPM, quinolone (CIP, MXF), DOX, SXT; 3 surgery	Prior macrolide, 10 (IQR, 4–17) versus 18.7 (IQR, 11.2–39.8)	38.7 (IQR, 11.4–41.9)

a Antibiotic or drug abbreviations: AMK, amikacin; AZM, azithromycin; CIP, ciprofloxacin; CLO, clofazimine; CLR, clarithromycin; DOX, doxycycline; EMB, ethambutol; ERY, erythromycin; FOX, cefoxitin; IPM, imipenem; LVX, levofloxacin; MEM, meropenem; MIN, minocycline; MXF, moxifloxacin; SXT, trimethoprim-sulfamethoxazole; TGC, tigecycline.

b CF, cystic fibrosis; AAT, abnormal α-1 antitrypsin; CD, ciliary dyskinesia; NA, data not available.

c NSS, M. abscessus subspecies not specified.

d i.v., intravenous.

e IQR, interquartile range.

Table 2 shows that while amikacin was given to patients in all studies examined, the other accompanying drugs in the combination therapy varied widely. In addition, amikacin duration and doses varied from as short as 2 weeks of daily parenteral doses to as long as a year given intermittently via either parenteral, inhalational, or liposomal delivery. Similarly, the duration of the combination therapy and follow-up also varied between studies and even between regimens examined in the same study. The number of patients receiving macrolide-free regimens in intent-to-treat analyses was 120, that for macrolide-containing regimens as initial therapy was 755, and that for patients with refractory disease was 138 (Table 2; see also the supplemental material).

### Mortality outcome.

Death was inconsistently reported, and its effect size was considered a critical risk of bias (Table 1). Moreover, only 7 (37%) studies reported on this outcome. The pooled death incidence was 16.67% (95% confidence interval [CI], 10.49 to 24.56%) with macrolide-free regimens. With regard to macrolide-containing regimens as initial therapy, only 3 (16%) studies reported deaths, and the pooled incidence was 15% (95% CI, 11 to 20%) with an *I*^2^ value of 75%. For macrolide-containing regimens in refractory patients, only 2 (11%) studies reported deaths. The pooled incidence was 4% (95% CI, 0 to 9%) with an *I*^2^ value of 0%. Given the critical risk of bias, further analyses of death as an outcome were not pursued.

### Sustained sputum culture conversion.

Only a single study reported use of macrolide-free regimens in 120 patients. Ten of 120 patients attained sustained sputum conversion. This translates to a sputum conversion rate of 8.33% (95% CI, 4.07 to 14.79%).

**FIG 2:**
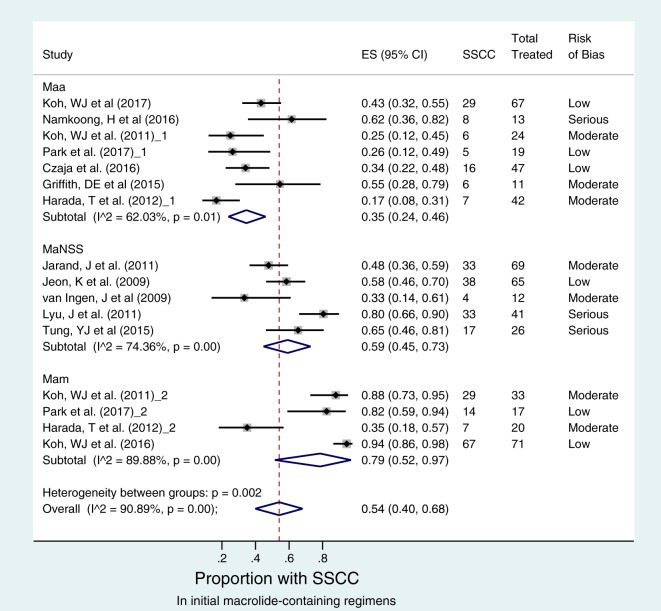
Sustained sputum culture conversion (SSCC) with initial macrolide-containing regimens. The forest plot depicts 13 studies comprising 16 macrolide-containing regimens that were examined as initial therapy in 223 treatment-naive patients with M. abscessus subsp. abscessus (designated Maa), 141 treatment-naive patients with M. abscessus subsp. massiliense (designated Mam), and 213 treatment-naive patients with M. abscessus with no subspecies specified (designated MaNSS). Risk of bias assessed for each effect size (ES) estimate is shown in the extreme right column. Despite the marked heterogeneity between these regimens (overall *I*^2^ value of >90%), patients with M. abscessus subsp. abscessus were significantly less likely to have SSCC than patients with M. abscessus subsp. massiliense, as shown by noninterloping confidence intervals between the two subspecies.

Regarding to macrolide-containing regimens as the initial therapy, there was significant heterogeneity among the 13 studies that examined sustained sputum culture (*I*^2^ = 91%; *P* < 0.001). However, there was lower heterogeneity among the seven M. abscessus subsp. abscessus disease studies (*I*^2^ = 62%; *P* = 0.01) (Fig. 2). There was no significant publication bias or small-study effects (*P* > 0.346 by Egger's test), suggesting that effect estimates from studies reflect true observations in study patients (Fig. S2). The forest plot in Fig. 2 shows that 77/223 patients with M. abscessus subsp. abscessus attained sustained sputum conversion, which was significantly lower than the 117/141 patients with M. abscessus subsp. massiliense disease. The sputum conversion rates were 35% (95% CI, 24 to 46%) in M. abscessus subsp. abscessus patients and 79% (95% CI, 52 to 97%) in M. abscessus subsp. massiliense patients. The odds ratio (OR) of sustained sputum conversion in M. abscessus subsp. abscessus versus M. abscessus subsp. massiliense diseases was 0.108 (95% CI, 0.066 to 0.181) (*P* < 0.001).

**FIG 3:**
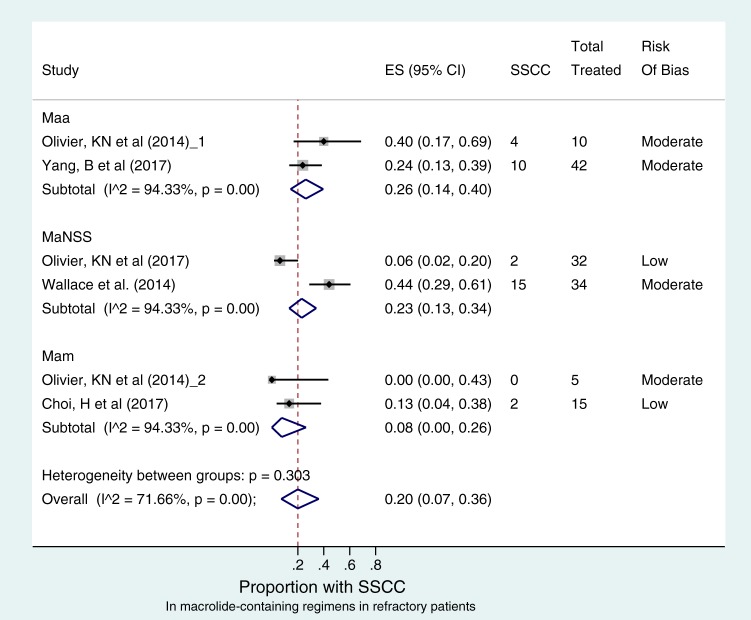
Sustained sputum conversion (SSCC) with macrolide-containing regimens in refractory patients. The forest plot depicts 5 studies comprising 6 macrolide-containing regimens that were examined in 52 refractory patients with M. abscessus subsp. abscessus, 20 refractory patients with M. abscessus subsp. massiliense, and 66 treatment-naive patients with M. abscessus with no subspecies specified. Risk of bias assessed for each effect estimate is shown in the extreme right column. There was no significant difference in SSCC between the subspecies. There was also marked heterogeneity in SSCC estimate across the different regimens (overall *I*^2^ value of 72%; *P* < 0.001).

The *I*^2^ value among the 5 studies that examined sustained sputum culture of macrolide-containing regimens in refractory patients was 72% (*P* < 0.001), indicating significant heterogeneity. However, there was no significant publication bias or small-study effects (*P* > 0.399) (Fig. S3). Figure 3 shows that only 33/138 (23%) patients attained sustained sputum conversion across all M. abscessus species. The pooled culture conversion rate was 20% (95% CI, 7 to 36%), which on follow-up after stopping therapy for 12 months was not significantly different across the mycobacterial species. Comparison between M. abscessus subsp. *abscessus*- and M. abscessus subsp. massiliense-infected patients revealed no statistical difference in sputum culture conversion: the odds ratio was 3.316 (95% CI, 0.680 to 15.74), and the *P* value was 0.205. New investigational drugs and novel delivery systems were applied in these combination regimens. The pooled sputum conversion rates in patients with M. abscessus with no subspecies specified after liposomal aminoglycoside therapy versus parenteral macrolide-containing therapy were 13.33% versus 5.88% (*P* = 0.589). Nonetheless, these results revealed that sputum culture conversion in refractory patients was very poor across all species and statistically not different from each other, regardless of route of administration of aminoglycosides.

### Recurrence after completing therapy.

**FIG 4:**
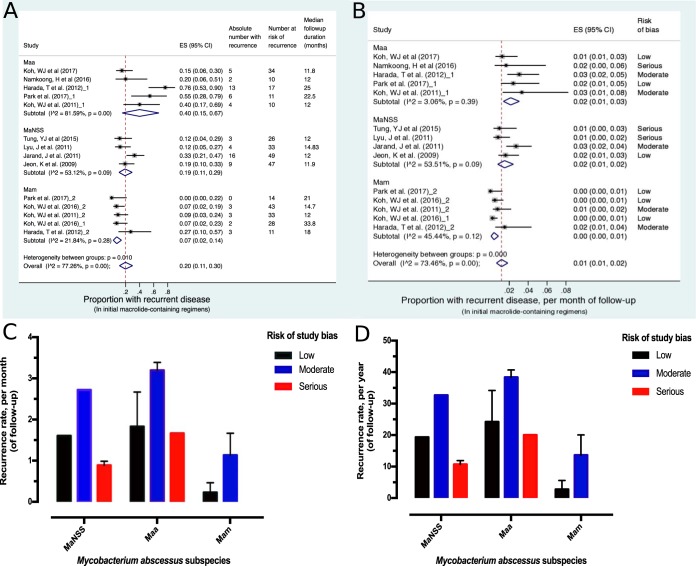
Recurrent pulmonary disease with confirmed M. abscessus complex. The forest plot depicts 10 studies comprising 14 macrolide-containing regimens that were examined after follow-up of 30 patients with M. abscessus subsp. abscessus, 11 patients with M. abscessus subsp. massiliense, and 32 patients with M. abscessus with no subspecies specified. Three hundred sixty-six patients were followed up and were at risk of recurrence; 73 suffered a recurrence. The median follow-up duration for each regimen is shown in the extreme right column in panel A, while the risk of bias is shown in the extreme right column in panel B. Panel A shows that, despite the marked heterogeneity between these regimens (overall *I*^2^ value of >77%), patients with M. abscessus subsp. abscessus were significantly more likely to have recurrent disease on follow-up, 40% (95% CI, 15 to 67%) compared to 7% (95% CI, 2 to 14%) in patients with M. abscessus subsp. massiliense, as shown by noninterloping confidence intervals between the two subspecies. The findings remain the same when the different follow-up durations are adjusted for, as shown in panel B. Panel C gives the average recurrence rate per month of follow-up, while panel D gives the same estimate per year of follow-up. Panels C and D also show that disease recurrences were significantly higher in studies with low/moderate risk of bias than those with some serious risk across the M. abscessus species.

Figure 4 shows forest plots of disease recurrence on follow-up after completing initial macrolide-containing therapy; these patients had initially responded to therapy. The analyses is based on recurrence in only 73 (20%) patients who were monitored for a mean of 16.68 (standard deviation [SD], 6.44) months in the 10 studies that reported these data (Table 2). The *I*^2^ value was 77% (*P* < 0.001), indicating significant heterogeneity between the studies. There was also significant bias (*P* = 0.030 by Egger's test) observed with the recurrence outcome studies. However, this bias was expected, since fewer studies (10/19) with even fewer patients reported on this outcome. Overall, disease recurrence in M. abscessus subsp. *abscessus*-infected patients was 40% (95% CI, 15 to 67%) versus 7% (95% CI, 2 to 14%) in M. abscessus subsp. massiliense-infected patients (Fig. 4A). The odds ratio of recurrence in M. abscessus subsp. abscessus-infected versus M. abscessus subsp. massiliense-infected patients was 6.189 (95% CI, 2.317 to 8.046). Since the follow-up duration was variable between studies and the risk of recurrence was significantly different between the isolated organisms, we adjusted for both and then expressed disease recurrence per month and per year of follow-up, as shown in Fig. 4B to D. The estimated recurrence rates were 1.835% (range, 1.667 to 3.196%) per month in M. abscessus subsp. abscessus disease and 0.683% (range, 0.229 to 1.136%) per month in M. abscessus subsp. massiliense disease. Thus, recurrence was significantly higher with M. abscessus subsp. abscessus infection across all studies regardless of the risk of bias of each study.

### Composite good versus poor outcomes.

Finally, in patients with appropriate follow-up, we defined good outcomes as sustained sputum conversion rate without relapse, a composite outcome, while the alternative was a poor outcome. The proportion of patients with good outcome was 52/223 (23%) with M. abscessus subsp. abscessus versus 118/141 (84%) with M. abscessus subsp. massiliense disease. The odds ratio of good outcomes was 0.059 (95% CI, 0.034 to 0.101) (*P* < 0.001).

### Sensitivity analyses.

Sensitivity analysis revealed that pooled sustained sputum culture conversion in M. abscessus complex disease was 48% (95% CI, 36 to 59%) in North American patients versus 68% (95% CI, 53 to 81%) in the Northeast Asian patients. The odds ratio for sputum conversion rate among North America patients was 0.458 (95% CI, 0.249 to 0.831) compared to Northeast Asia patients (*P* = 0.015). However, when analysis was restricted to patients with M. abscessus subsp. abscessus infection, pooled sputum culture conversions were 37% (95% CI, 25 to 51%) in North America versus 33% (95% CI, 19 to 48%) in Northeast Asia. The odds ratio was 1.222 (95% CI, 0.648 to 2.312) and not significantly different (*P* = 0.526). This suggests that differences in sputum conversion rates between the two locales are partially driven by differences in prevalence of infection with different subspecies. Finally, we also examined the differential effect of macrolides (clarithromycin versus azithromycin) on sputum culture conversion and recurrence. There was no significant difference between the two drugs for either organism (Fig. S5). Risk of bias did not change our findings even after metaregression analyses, shown in Fig. S6.

## DISCUSSION

In the treatment of drug-susceptible tuberculosis (TB), more than 80% of patients respond to therapy. For the much-feared multidrug-resistant TB (MDR-TB), microbial response rates are 50 to 90%; for the dreaded extensively drug-resistant TB (XDR-TB), favorable outcomes are found for 16% of patients at 24 months of follow-up (35). Here, we show that outcomes in patients with presumed drug-susceptible M. abscessus subsp. abscessus are dramatically worse than those for patients with MDR-TB and similar to those for patients with XDR-TB. Outcomes in patients with M. abscessus subsp. massiliense pulmonary disease were also poor but similar to those for patients with MDR-TB. In fact, we found outcomes are significantly worse than cure rates of >95% and 10% relapse over 10 years for the epic and ancient disease of leprosy (36). In a recent meta-analysis, we found that the cure rates for pulmonary Mycobacterium avium complex (MAC) were roughly 50%, which is very poor but still better than that for M. abscessus subsp. abscessus disease (37). Thus, the main finding is that we can say with confidence that M. abscessus complex pulmonary disease outcomes on modern chemotherapy are currently the worst for all mycobacterial species and are atrocious.

The general notion is that macrolides improve outcomes in M. abscessus complex, perhaps based on outcomes in MAC. We show that even with macrolide regimens, outcomes in pulmonary M. abscessus complex are very poor. Recently, in a hollow-fiber pharmacokinetics/pharmacodynamics (PK/PD) model of M. abscessus subsp. abscessus disease, we demonstrated that even when drug concentrations were at their most optimal, the standard macrolide-containing regimen had poor maximal microbial kill (*E*_max_) (9, 13). At *E*_max_, all antibiotic target sites are saturated or bound by antibiotic; thus, increasing the antibiotic concentration or dose will not result in increased kill (i.e., *E*_max_ is fixed for a drug or combination). Thus, it was not surprising that even inhalational therapy did not improve outcomes, since regimens cannot kill any more than their *E*_max_ (9, 13). Indeed, in the PK/PD studies ADR arose on clarithromycin, amikacin, and cefoxitin combination therapy even at the *E*_max_ of each drug in the regimen. Our meta-analysis findings are consistent with the PK/PD work and suggest that the currently recommended regimen for M. abscessus subsp. abscessus lung disease has limited to no value, regardless of method of delivery of the drugs.

We propose that the hope that macrolides could improve outcomes was partially based on the misguided idea of classifying highly virulent mycobacterial species as nontuberculous mycobacteria (NTM), equivalent to classifying lions and elephants in the African savanna as non-hyena animals. This led to conflation of improvement of different mycobacterial species (NTM) responses to macrolides with M. abscessus complex, which obviously do not respond. Our results show that there is no therapeutic benefit to conflating different mycobacterial species, even within M. abscessus complex itself: M. abscessus subsp. massiliense had dramatically different response rates than M. abscessus subsp. abscessus. Therefore, why conflate them or combine them in the same category? It is hoped that in the era of WGS and matrix-assisted laser desorption ionization–time-of-flight mass spectrometry, the designation of non-hyena animals will be ditched. No one ever calls a Shigella species a non-E. coli organism, even though Shigella species are considered to belong to the genus Escherichia in the Enterobacteriaceae family, a closer relationship than M. avium to M. abscessus subsp. abscessus. Therapies for M. abscessus subsp. abscessus and M. abscessus subsp. massiliense should be sought without reference to M. tuberculosis or even M. avium, which in any case have much more favorable outcomes than their cousins.

There are several strengths and limitations to our study. First, as stated throughout our results, there was much heterogeneity of studies. However, we used a validated instrument to assess the risk of bias and then applied random effects models with subgroup analyses in anticipation of much heterogeneity. Metaregression methods and sensitivity analyses supported this approach by consistently showing that our designated subgroups were homogenous, and the same estimates were obtained with these different approaches. This suggests that our estimates are robust and that our conclusions will be reproduced in future studies. A second important limitation is that the same authors from the same institutions performed a considerable number of the retrospective studies, albeit with different enrollment time frames and inclusion criterion but nonetheless drawing from the same databases. While we were able to exclude obvious duplicate studies of the same patient cohorts, in some instances we could have failed to decipher whether the same patients were reported in different studies. The third limitation relates to differential sputum sampling between patients within and between studies for both diagnosis and monitoring disease during therapy. Sputum sampling did not follow an identical schedule between studies. Fourth, the decision to treat pulmonary M. abscessus complex is based on the balance of potential risks and benefits for each individual. Patients who opted not to be treated even with severe disease were not included in the analyses, suggesting that the atrocious outcomes that were reported and we identified are more optimistic than findings in the clinic. As an example, our estimates of pooled mortality estimates are subject to critical risk of bias, while those for disease recurrence have moderate risk of bias.

## MATERIALS AND METHODS

### Search strategy.

We followed the PRISMA guidelines in performing and reporting the systematic review and meta-analyses (38). The following inclusion criteria were used for study selection: (i) American Thoracic Society (ATS) and Infectious Diseases Society of America (IDSA) bacteriological criteria for making diagnosis of pulmonary M. abscessus complex disease or infection, (ii) clear specification of combination therapy received by patients, and (iii) clear specification of microbial outcomes attained by patients after 12 months of treatment or at the end of treatment. Studies judged to be at (i) critical risk for bias or (ii) without adequate information upon which risk of bias for any of the outcomes evaluated in that study could be made were excluded from the meta-analyses. There is no consensus on definitions for treatment outcomes in nontuberculous mycobacterial pulmonary diseases, so we used the outcomes (including sputum culture conversion) stated by each study to define composite outcomes. Most studies defined sputum conversion as 2 to 3 or more consecutive negative cultures, consistent with ATS and IDSA criteria (6). If a patient failed to expectorate sputum, then the sputum was considered to have converted to negative. Sustained sputum culture conversion (SSCC) denotes patients who converted to negative and did not relapse during therapy. Sputum relapse and failure to convert sputum to culture negative with 12 months of therapy was considered treatment failure. Death from any cause was also considered treatment failure. Thus, in this study the term “recurrence” was used to define either disease relapse with the same isolates or disease reinfection with different isolates after sputum culture conversion, since earlier studies did not perform M. abscessus complex isolate identification and comparison.

The complete search strategy used with each database is included in the supplemental material. Briefly, we searched PubMed and Embase for reports published before 30 March 2017 that included MeSH terms or the free-text terms “nontuberculous mycobacteria,” “rapid growing mycobacteria,” “Mycobacterium abscessus,” “Mycobacterium abscessus complex,” “Mycobacterium massiliense,” or “Mycobacterium bolletii.” The search terms were combined with the MeSH terms “treatment” or “therapy” and “outcomes.” There were no language restrictions. The computer search was also supplemented by going through the list of references of systematic reviews on the subject and a search of the Grey Literature Database (http://www.greylit.org/).

Each study was examined for systematic bias using the ROBINS-I tool (version 1.0.0; www.riskofbias.info). The ROBIN-I tool addresses several weaknesses identified in previous instruments used to measure study quality in observational studies, is easy to use, is easy to interpret, and is highly reproducible (39–41). The ROBINS-I tool measures bias for each effect size in seven domains: confounding, selection of participants, classification of interventions, departure from intended interventions, missing data, measurement of outcomes, and selection of reported outcomes (40). Each study had an overall risk of bias judgment calculated across all seven domains and was graded into one of five levels of risk: low, moderate, serious, critical, and no information. Three effect sizes or microbial outcomes were assessed for risk of bias: death, therapy success (i.e., sustained sputum conversion), and disease recurrence. Critical risk of bias referred to serious risk of bias in two or more domains. Such high levels of bias were considered to lead to imprecision in effect size estimation. Therefore, studies with critical risk of bias or no information available to determine level of risk were excluded from the meta-analysis. Similarly, case reports or studies restricted to patients with specific pulmonary clinical conditions, such as pre- or posttransplant or cystic fibrosis, were excluded.

### Data abstraction.

The following data were extracted from each study: (i) the author(s) and the year the study was conducted and published, (ii) criteria used to establish pulmonary M. abscessus complex disease, (iii) number of patients enrolled, receiving therapy, and had outcomes evaluated, (iv) combination therapy regimens examined and duration of therapy, and (v) number of patients with outcomes. Data were extracted from tables, text, or referred articles. Two reviewers (J.G.P. and D.O.) independently examined the studies for bias and extracted the data into a prespecified electronic database. The two databases were compared for consistency, and disagreements were settled after consultation with a third reviewer (T.G.). ATS/IDSA standard definitions and terms were used throughout (6).

### Statistical analysis.

We used meta-analysis methods to estimate the proportion of patients with the following outcomes: SSCC and disease recurrence after at least 12 months of therapy. These definitions are consistent with ATS/IDSA therapy targets for microbiologic outcomes during treatment of nontuberculous mycobacterial infections. We computed odds ratios (OR) and their 95% confidence intervals (CI) across therapy regimens, stratified by organism and geographic locale. Since macrolides are considered essential for M. abscessus complex therapy (6), regimens were grouped into one of three categories: (i) macrolide-free regimens, (ii) macrolide-containing regimens used as the initial therapy for treatment-naive patients, or (iii) macrolide-containing regimens used in patients with refractory pulmonary disease. In the analyses, the term “Mycobacterium abscessus no species specified” was reserved for patients who did not have the subspecies characterized or had mixed infections.

The DerSimonian and Laird random effects model, which incorporates variation between studies in weighting, was used to pool estimates and performed with STATA software, version 14 (College Station, TX). Freeman and Tukey double arcsine transformation was used to stabilize the variance (42). This allowed identification of admissible 95% CI in events when sample sizes were small and/or proportions were near the margins. We used the *I*^2^ statistic to quantify heterogeneity of the effect size estimates between patient groups and between studies (39). Sensitivity analyses and metaregression were used to assess the veracity of findings (43). Egger's test was used to assess for publication bias and small-study effects.

## Supplementary Material

Supplemental material
