# Supplementary material for: Systematic Review and Meta-analyses of the Effect of Chemotherapy on Pulmonary Mycobacterium abscessus Outcomes and Disease Recurrence
Source: Antimicrob Agents Chemother. 2017 Oct 24;61(11):e01206-17. doi: 10.1128/AAC.01206-17 (PMC5655093; doi:10.1128/AAC.01206-17)
Supplement: Supplemental material [file supp_61_11_e01206-17__index.html]

Supplemental material 

# Systematic Review and Meta-analyses of the Effect of Chemotherapy on Pulmonary Mycobacterium abscessus Outcomes and Disease Recurrence

## Supplemental material

- Supplemental file 1 -

  Fig. S1 to S6 and supplemental text

  PDF, 328K
