# Supplementary material for: Systematic Review and Meta-analyses of the Effect of Chemotherapy on Pulmonary Mycobacterium abscessus Outcomes and Disease Recurrence
Source: Antimicrob Agents Chemother. 2017 Oct 24;61(11):e01206-17. doi: 10.1128/AAC.01206-17 (PMC5655093; doi:10.1128/AAC.01206-17)
Supplement: Supplemental material [file AAC.01206-17_zac011176607s1.pdf]

Supplemental methods and results

Systematic review and meta-analyses of the effect of chemotherapy on pulmonary *Mycobacterium abscessus* outcomes and disease recurrence

Jotam G. Pasipanodya[1], Deborah Ogbonna[1], Beatrix E. Ferro[2], Gesham Magombedze[1], ShashiKanti Srivastava[1], Devyani Deshpande[1], Tawanda Gumbo[1, 3]

1. Center for Infectious Diseases Research and Experimental Therapeutics (CIDRET), Baylor Research Institute, 3434 Live Oak Street, Dallas, 75204, Texas
2. Universidad ICESI
3. Department of Medicine, University of Cape Town, Observatory, Cape Town, South Africa.

Corresponding Author,
Tawanda Gumbo, MD
CIDRET, Baylor University Medical Center, 3434 Live Oak Street,
Dallas, 75204, Texas, USA
Phone: 214 820 9561
Fax: 214 820 4813
Email: Tawanda.gumbo@BSWHealth.org

Contents **Page**

Search strategy	1
Supplemental Figure S1	2
Supplemental Figure S2	3
Supplemental Figure S3	4
Supplemental Figure S4	5
Supplemental Figure S5	6 &7
Supplemental Figure S6	8-10

Supplemental methods

Search strategy in PubMed, EMBASE and Grey Literature

"*Mycobacterium massiliense*"[All Fields] OR "*Mycobacterium abscessus*"[All Fields] OR "*Mycobacterium bolletii*"[All Fields]

AND

(("therapy"[Subheading] OR "therapy"[All Fields] OR "therapeutics"[MeSH Terms] OR "therapeutics"[All Fields]) AND ("therapy"[Subheading] OR "therapy"[All Fields] OR "treatment"[All Fields] OR "therapeutics"[MeSH Terms] OR "therapeutics"[All Fields]))

AND

(outcome [All Fields] OR outcomes [All Fields])

Variations of the above were used as free text in EMBASE and the Grey literature, with Boolean combinations used.

Supplemental results

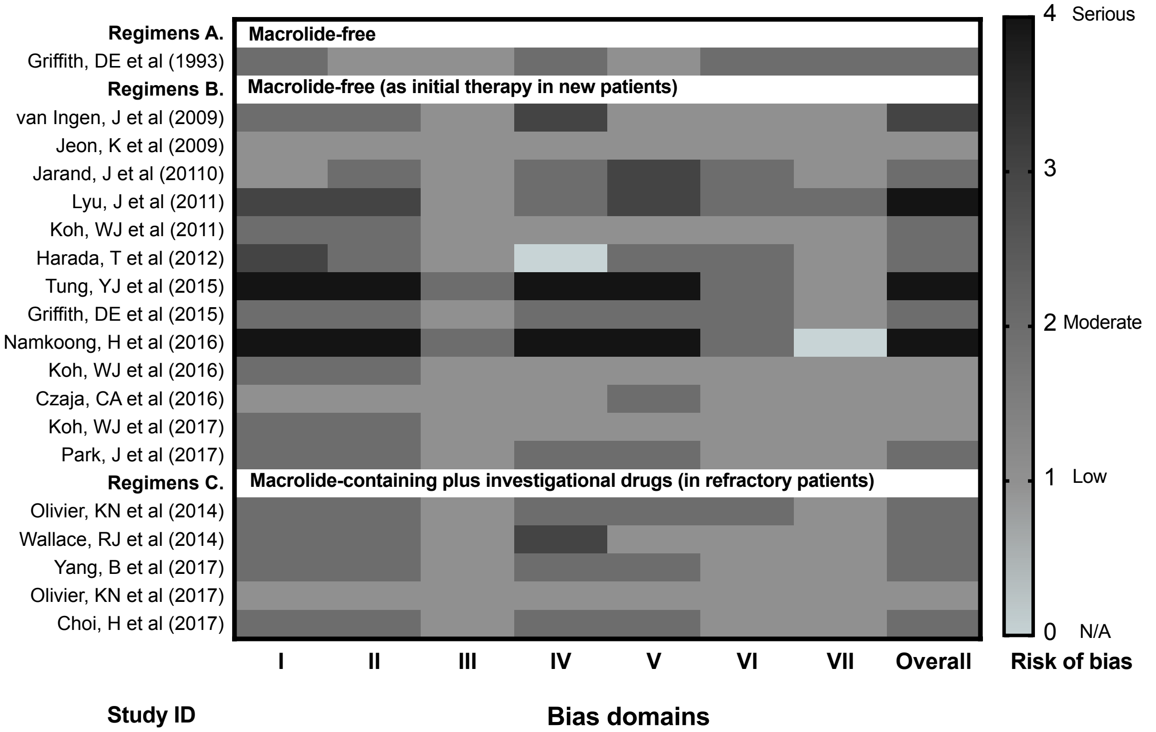

Supplemental Figure S1 Risk of bias in selected studies

Supplemental Figure S1 shows is a heat map showing changes in risk of bias within each study across the VII domains tested for bias. Lighter shades indicate low risk of bias and darker shades indicate higher risk of bias.

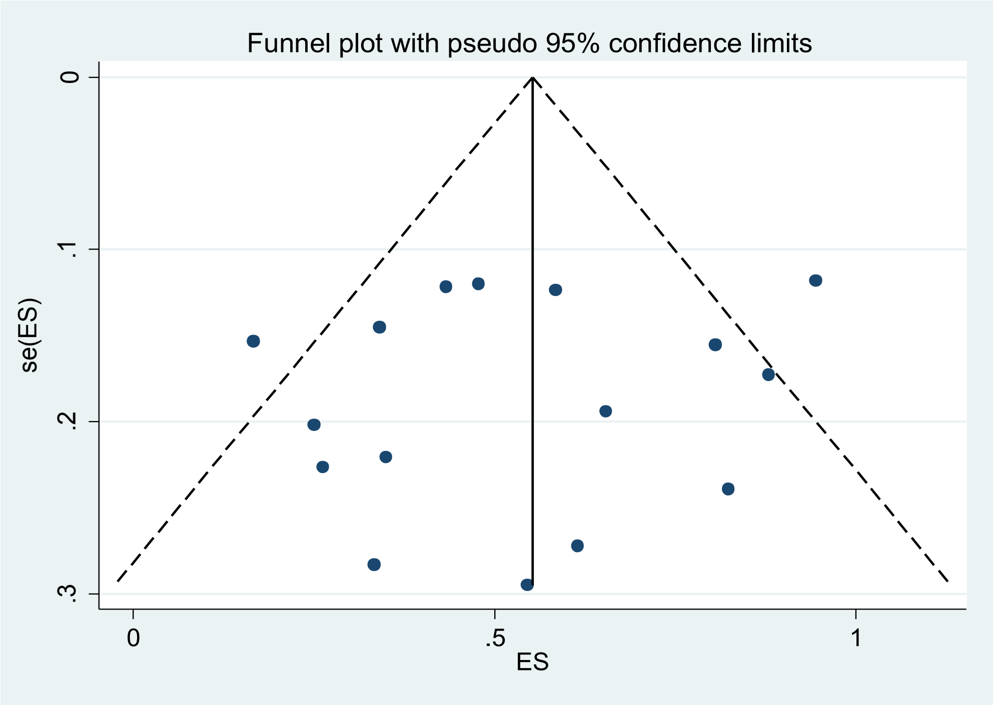

Supplemental Figure S2. Publication bias and small study effect plot in initial macrolide-free regimens.

The precision in the estimation of effect increased as the size of each study increased; slope 0.68 (95% CI, 0.21-1.15), p=0.008. The null hypothesis for Egger's test is that symmetry exists in the funnel plot, with the alternative indicating that asymmetry is present. The p-value for Egger's test in this case was 0.541, indicating that there was no apparent bias in the meta-analysis

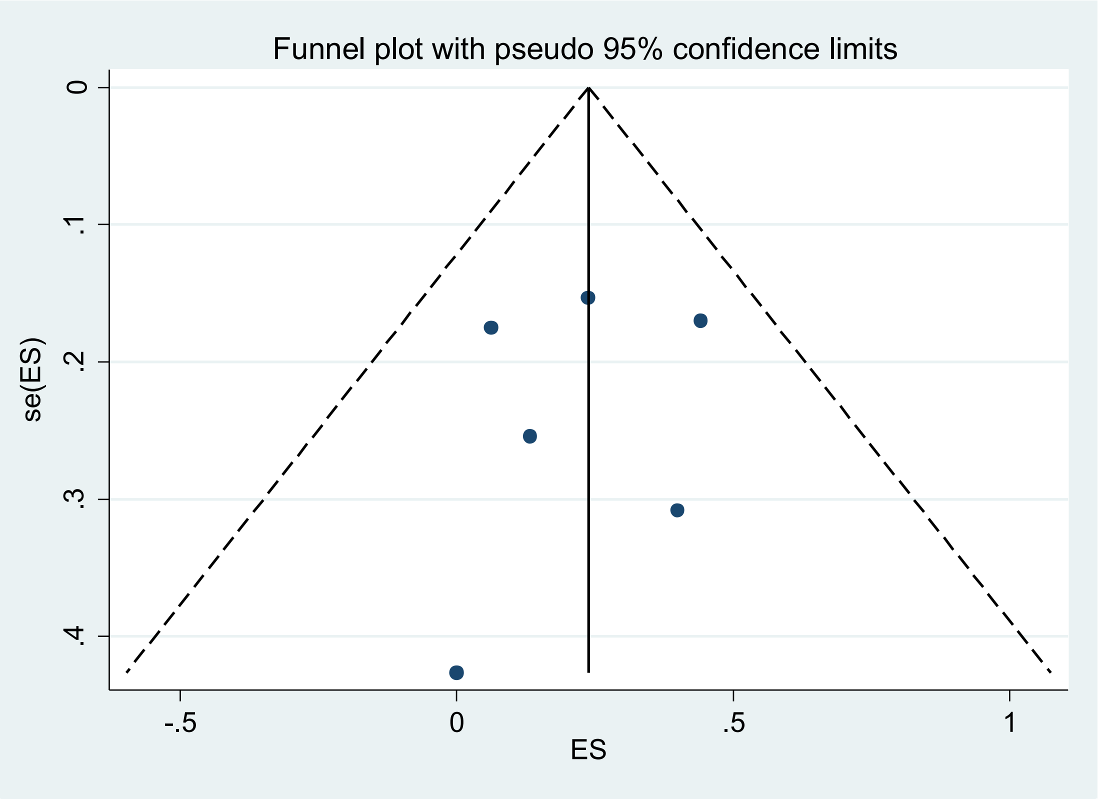

Supplemental Figure S3. Publication bias and small study effect plot in macrolide-containing regimens in refractory pulmonary Mycobacterium abscessus patients.

The slope was 0.33 (95% CI, -0.32-0.99), p=0.236. Egger test for small study effect revealed p-value of 0.711; however, the small number of studies (5) and marked heterogeneity potentially biases the Egger tests.

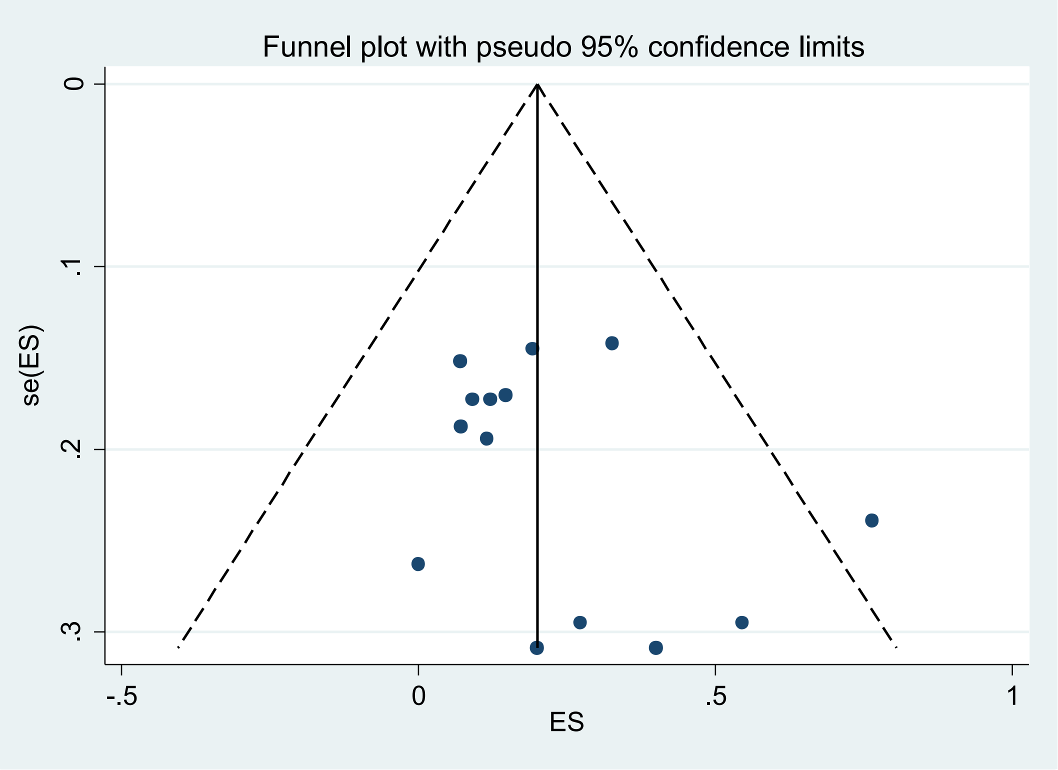

Supplemental Figure S4. Publication bias and small study effect plot of disease recurrence.

The slope was -0.01 (95% CI, -0.40-0.37), p=0.236. Egger test for small study effect revealed p-value of 0.233; however, significant heterogeneity potentially biases the Egger tests.

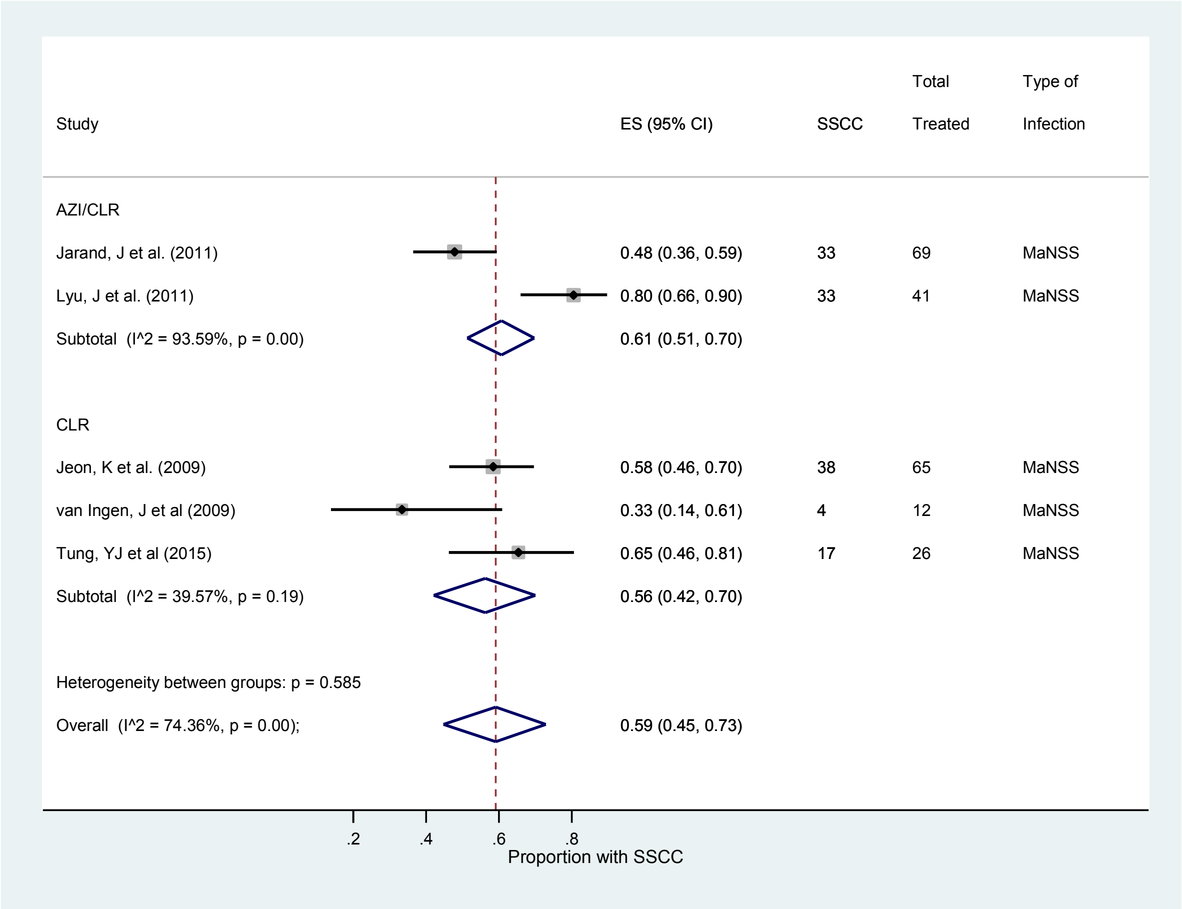

Supplemental Figure S5 Comparison of macrolides across Mycobacterial abscessus species.

The forest plot in **Figure S5A** shows that there was no significant difference in sustained sputum conversion (SSCC) in patients with *Mycobacterium abscessus* no species specified (MaNSS) between those treated with clarithromycin regimens compared to those treated with azithromycin/clarithromycin.

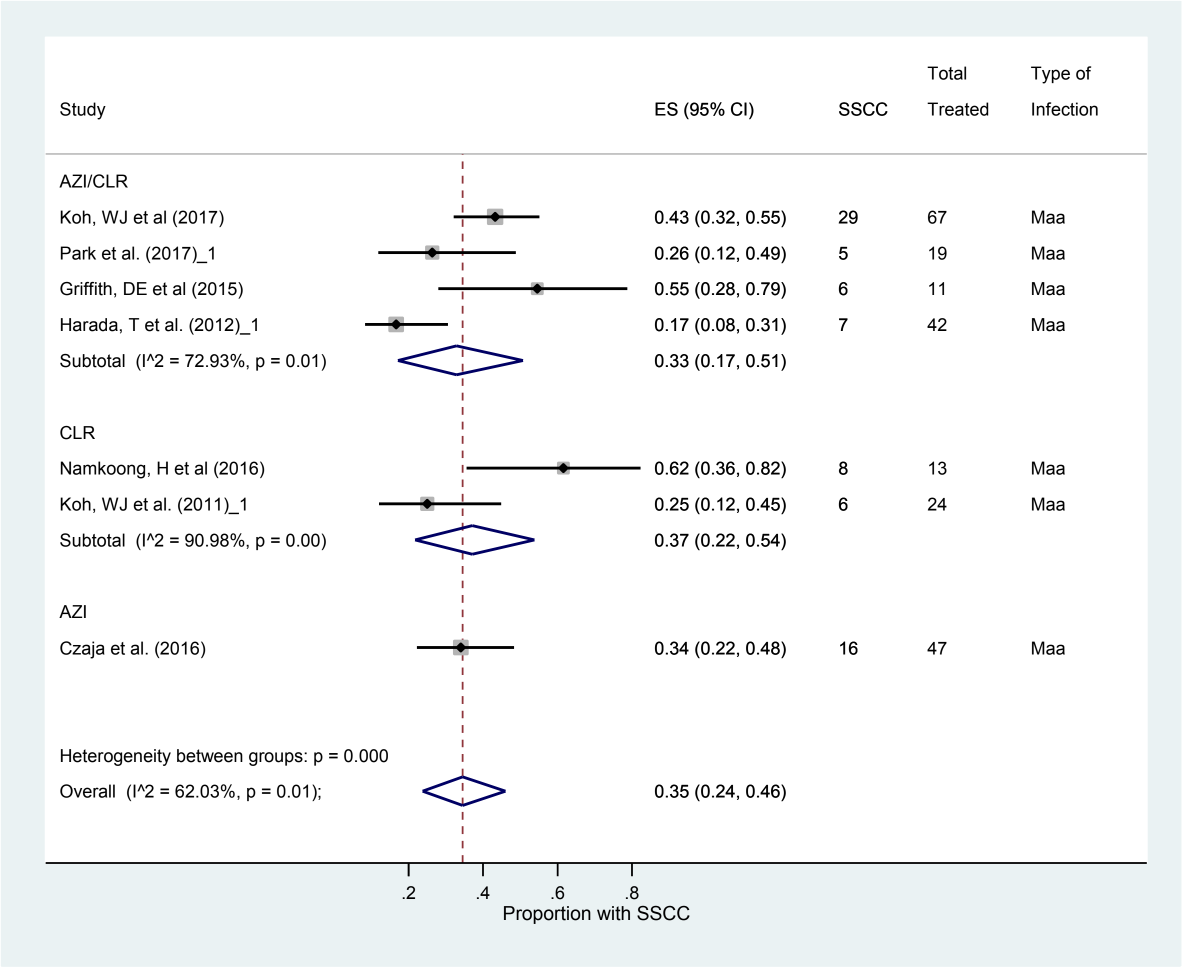

Supplemental Figure S5 Comparison of macrolides across Mycobacterial abscessus species.

The forest plot in **Figure S5B** shows that there was no significant difference in sustained sputum conversion (SSCC) in patients with *Mycobacterium abscessus* subspecies *abscessus* (Maa) between those treated with clarithromycin regimens compared to those treated with azithromycin/clarithromycin. Supplementary Figure S5A Comparison of clarithromycin versus azithromycin/clarithromycin

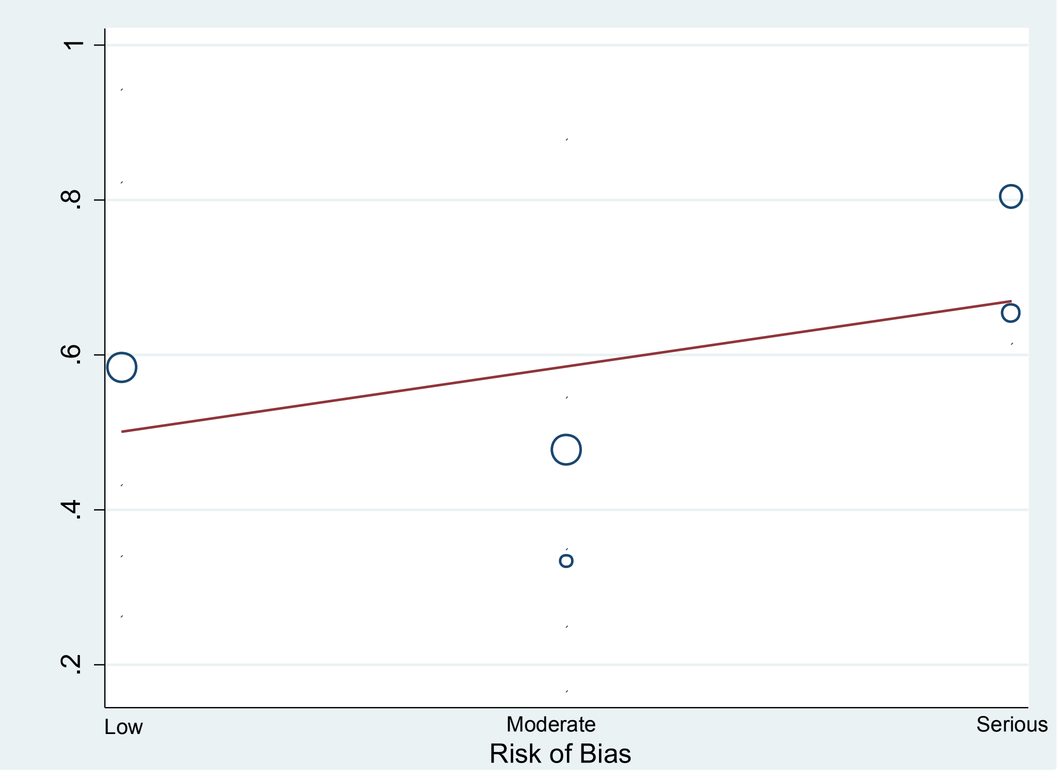

Supplemental Figure S6 Metaregression of the effect of risk of bias on sustained sputum conversion.

Figure S6A shows that there was no significant association between SSCC and risk of bias when the size of the study was adjusted for: estimate for the slope was 0.08 (95% CI, -0.21-0.37), p=0.426.

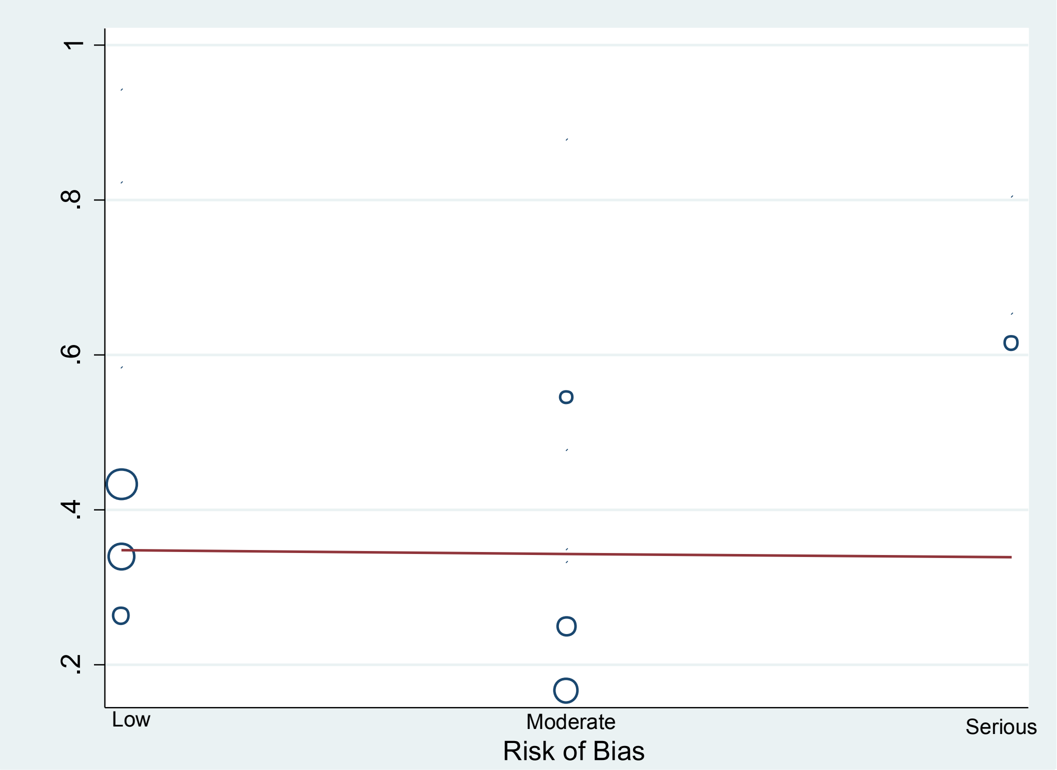

Supplemental Figure S6 Metaregression of the effect of risk of bias on sustained sputum conversion.

Figure S6B shows that there was no significant association between SSCC and risk of bias when the size of the study was adjusted for: estimate for the slope was 0.00 (95% CI, -0.29-0.28), p=0.969.

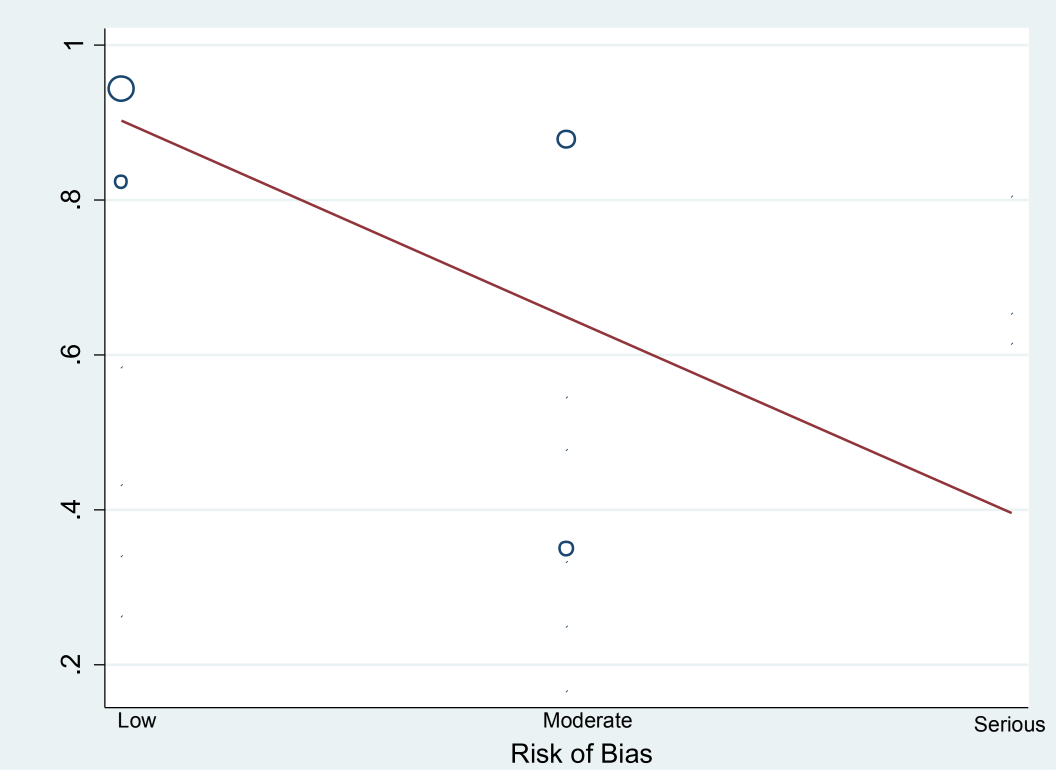

Supplemental Figure S6 Metaregression of the effect of risk of bias on sustained sputum conversion.

Figure S6C shows that there was no significant association between SSCC and risk of bias when the size of the study was adjusted for: estimate for the slope was -0.25 (95% CI, -1.37-0.87), p=0.433.

Supplemental references

1. **Griffith, D. E., W. M. Girard, and R. J. Wallace, Jr.** 1993. Clinical features of pulmonary disease caused by rapidly growing mycobacteria. An analysis of 154 patients. Am Rev Respir Dis **147**:1271-1278.
2. **van Ingen, J., Z. R. de Zwaan, R. P. Dekhuijzen, M. J. Boeree, and S. D. van Soolingen**. 2009. Clinical relevance of *Mycobacterium chelonae-abscessus* group isolation in 95 patients. J Infect **59**:324-331.
3. **Jeon, K., O. J. Kwon, N. Y. Lee, B. J. Kim, Y. H. Kook, S. H. Lee, Y. K. Park, C. K. Kim, and W. J. Koh**. 2009. Antibiotic treatment of *Mycobacterium abscessus* lung disease: a retrospective analysis of 65 patients. Am J Respir Crit Care Med **180**:896-902.
4. **Jarand, J., A. Levin, L. Zhang, G. Huitt, J. D. Mitchell, and C. L. Daley**. 2011. Clinical and microbiologic outcomes in patients receiving treatment for *Mycobacterium abscessus* pulmonary disease. Clin Infect Dis **52**:565-571.
5. **Lyu, J., H. J. Jang, J. W. Song, C. M. Choi, Y. M. Oh, S. D. Lee, W. S. Kim, D. S. Kim, and T. S. Shim**. 2011. Outcomes in patients with *Mycobacterium abscessus* pulmonary disease treated with long-term injectable drugs. Respir Med **105**:781-787.
6. **Koh, W. J., K. Jeon, N. Y. Lee, B. J. Kim, Y. H. Kook, S. H. Lee, Y. K. Park, C. K. Kim, S. J. Shin, G. A. Huitt, C. L. Daley, and O. J. Kwon**. 2011. Clinical significance of differentiation of *Mycobacterium massiliense* from Mycobacterium abscessus. Am J Respir Crit Care Med **183**:405-410.
7. **Harada, T., Y. Akiyama, A. Kurashima, H. Nagai, K. Tsuyuguchi, T. Fujii, S. Yano, E. Shigeto, T. Kuraoka, A. Kajiki, Y. Kobashi, F. Kokubu, A. Sato, S. Yoshida, T. Iwamoto, and H. Saito**. 2012. Clinical and microbiological differences between *Mycobacterium abscessus* and *Mycobacterium massiliense* lung diseases. J Clin Microbiol **50**:3556-3561.
8. **Tung, Y. J., S. O. Bittaye, J. R. Tsai, C. Y. Lin, C. H. Huang, T. C. Chen, W. R. Lin, K. Chang, C. C. Lai, P. L. Lu, and Y. H. Chen**. 2015. Risk factors for microbiologic failure among Taiwanese adults with *Mycobacterium abscessus* complex pulmonary disease. J Microbiol Immunol Infect **48**:437-445.
9. **Griffith, D. E., J. V. Philley, B. A. Brown-Elliott, J. L. Benwill, S. Shepherd, D. York, and R. J. Wallace, Jr.** 2015. The significance of *Mycobacterium abscessus* subspecies *abscessus* isolation during *Mycobacterium avium* complex lung disease therapy. Chest **147**:1369-1375.
10. **Namkoong, H., K. Morimoto, T. Nishimura, H. Tanaka, H. Sugiura, Y. Yamada, A. Kurosaki, T. Asakura, S. Suzuki, H. Fujiwara, K. Yagi, M. Ishii, S. Tasaka, T. Betsuyaku, Y. Hoshino, A. Kurashima, and N. Hasegawa**. 2016. Clinical efficacy and safety of multidrug therapy including thrice weekly intravenous amikacin administration for *Mycobacterium abscessus* pulmonary disease in outpatient settings: a case series. BMC Infect Dis **16**:396.
11. **Koh, W. J., B. H. Jeong, K. Jeon, S. Y. Kim, K. U. Park, H. Y. Park, H. J. Huh, C. S. Ki, N. Y. Lee, S. H. Lee, C. K. Kim, C. L. Daley, S. J. Shin, H. Kim, and O. J. Kwon**. 2016. Oral Macrolide Therapy Following Short-term

Combination Antibiotic Treatment of *Mycobacterium massiliense* Lung Disease. Chest **150**:1211-1221.

12. **Czaja, C. A., A. R. Levin, C. W. Cox, D. Vargas, C. L. Daley, and G. R. Cott**. 2016. Improvement in Quality of Life after Therapy for *Mycobacterium abscessus* Group Lung Infection. A Prospective Cohort Study. Ann Am Thorac Soc. **13**:40-48.
13. **Koh, W. J., B. H. Jeong, S. Y. Kim, K. Jeon, K. U. Park, B. W. Jhun, H. Lee, H. Y. Park, D. H. Kim, H. J. Huh, C. S. Ki, N. Y. Lee, H. K. Kim, Y. S. Choi, J. Kim, S. H. Lee, C. K. Kim, S. J. Shin, C. L. Daley, H. Kim, and O. J. Kwon**. 2017. Mycobacterial Characteristics and Treatment Outcomes in *Mycobacterium abscessus* Lung Disease. Clin Infect Dis **64**:309-316.
14. **Park, J., J. Cho, C. H. Lee, S. K. Han, and J. J. Yim**. 2017. Progression and Treatment Outcomes of Lung Disease Caused by *Mycobacterium abscessus* and *Mycobacterium massiliense*. Clin Infect Dis **64**:301-308.
15. **Olivier, K. N., P. A. Shaw, T. S. Glaser, D. Bhattacharyya, M. Fleshner, C. C. Brewer, C. K. Zalewski, L. R. Folio, J. R. Siegelman, S. Shallom, I. K. Park, E. P. Sampaio, A. M. Zelazny, S. M. Holland, and D. R. Prevots**. 2014. Inhaled amikacin for treatment of refractory pulmonary nontuberculous mycobacterial disease. Ann Am Thorac Soc. **11**:30-35.
16. **Wallace, R. J., Jr., G. Dukart, B. A. Brown-Elliott, D. E. Griffith, E. G. Scerpella, and B. Marshall**. 2014. Clinical experience in 52 patients with tigecycline-containing regimens for salvage treatment of *Mycobacterium abscessus* and *Mycobacterium chelonae* infections. J Antimicrob Chemother. **69**:1945-1953.
17. **Yang, B., Jhun, B. W., Moon, S. M., Lee, H., Park, H. Y., Jeon, K., Kim, D. H., Kim, S. Y., Shin, S. J., Daley, C. L., and Koh, W. J. A.** 2017. Clofazimine-Containing Regimen for the Treatment of *Mycobacterium abscessus* Lung Disease. Antimicrob Agents Chemother **61**: e02052-16.
18. **Olivier, K. N., D. E. Griffith, G. Eagle, J. P. McGinnis, L. Micioni, K. Liu, C. L. Daley, K. L. Winthrop, S. Ruoss, D. J. Addrizzo-Harris, P. A. Flume, D. Dorgan, M. Salathe, B. A. Brown-Elliott, R. Gupta, and R. J. Wallace, Jr.** 2017. Randomized Trial of Liposomal Amikacin for Inhalation in Nontuberculous Mycobacterial Lung Disease. Am J Respir Crit Care Med **195**:814-823.
19. **Choi, H., S. Y. Kim, H. Lee, B. W. Jhun, H. Y. Park, K. Jeon, D. H. Kim, H. J. Huh, C. S. Ki, N. Y. Lee, S. H. Lee, S. J. Shin, C. L. Daley, and W. J. Koh**. 2017. Clinical Characteristics and Treatment Outcomes of Patients with Macrolide-Resistant *Mycobacterium massiliense* Lung Disease. Antimicrob Agents Chemother. **61**:e02189-16
